# Vaginal douching by women with vulvovaginitis and relation to reproductive health hazards

**DOI:** 10.1186/1472-6874-13-23

**Published:** 2013-05-14

**Authors:** Omar M Shaaban, Alaa Eldin A Youssef, Mostafa M Khodry, Sayed A Mostafa

**Affiliations:** 1 Department of Obstetrics and Gynecology, Faculty of Medicine, Assiut University, Assiut, Egypt

**Keywords:** Vaginal douching, Reproductive implications, Vaginal infections

## Abstract

**Background:**

Vaginal douching (VD) is a common practice among married women all over the world specially those in the Middle East. It is used for personal hygiene or for other aesthetic reasons in many countries. The current study investigates the prevalence of VD among patients with vulvovaginitis in Egypt. It also compares the reproductive health hazards among women performing routine VD with those using external hygiene. It also investigates why, and how women practice this douching.

**Methods:**

A cross sectional observational study was conducted in a tertiary university affiliated hospital in Assiut, Egypt. An interview administered questionnaire was administered to 620 women by two trained clinic nurses. Women presented to the outpatient clinic and diagnosed to have any type of vaginal infections were approached for participation. The principle outcome was the history of preterm labor in women who routinely performed VD versus those who did not (upon which sample size was estimated). Other outcome measures were the types of vaginal infections, and reproductive implications comprising, ectopic pregnancy, abortion and pelvic inflammatory disease (PID).

**Results:**

The participants were predominantly multiparas from semi-urban background and middle socioeconomic level. Considering VD as a religious duty and a kind of personal cleanliness were the most common reasons for performing VD in 88.9% and 80.6% of the studied population, respectively. History of preterm labor was reported in 19.2% versus 11.9% (p=0.048), while history of PID in 13.2% versus 6.0% (p=0.008) in women performing VD compared to those not performing this habit, respectively. There were no significant differences between the two groups as regard the history of ectopic pregnancy or the number of previous abortions.

**Conclusion:**

Vaginal douching is a prevalent practice in Egypt and has traditional and religious roots within the community. There are many misbeliefs around this habit in Egypt. Vaginal douching increases certain reproductive health hazards especially preterm labor and PID. Much effort and awareness campaigns are needed to increase women awareness about health hazards of this incorrect practice and to limit its use.

## Background

Vaginal douching (VD) is the process of intravaginal cleansing with any type of liquid solution [1,2]. Douching is a common practice among women all over the world and is used for personal hygiene or other aesthetic reasons in many countries [2,3]. Studies in the USA showed that about 37% of US women in reproductive age (15–44) reported regular VD. The performance of this habit varied between different ethnic groups and different socioeconomic levels [4]. In a Turkish study, VD reported to be performed by 91.6% of women living in rural areas [5].

There are cultural believes that VD is necessary for good hygiene. Other motives for douching are to clean the vagina after the end of menses or before and/or after sexual intercourse, to prevent or ameliorate an odor, to prevent or treat vaginal symptoms such as itching and discharge, and less commonly, to prevent pregnancy [6]. Other factors contribute to a woman's decision to douche her vagina like the influence of patients' mothers, friends, and relatives [5,7]. Some others consider VD as religious duty [to be able to pray) to purify their bodies after menses or sexual intercourse [8].

Previous studies have pointed to reproductive health hazards of VD. Women who perform this habit were 1.2 to 5.1 times more likely to develop bacterial vaginosis (BV) [9]; 1.6 -1.9 times more likely to experience a preterm labor (PTL) [10-12]. Women who adopt this habit were also almost 4 times at higher risk of ectopic pregnancy [13]; twice likely to develop cancer cervix; 1.7 times more likely to have sexually transmitted infections (STIs) [14]. Moreover, VD performers have 73% increased risk of pelvic inflammatory disease (PID) [2] and are 1.5 times more likely to develop endometritis [15].

The current study aims to determine the frequency of VD practice among women presenting to Assiut University Hospital Outpatient Clinic with any type of vulvovaginitis. The study also aims to determine if there is relation between the VD and the frequency of having vaginal infection and reproductive hazards, like, PID, abortion, ectopic pregnancy and PTL. Additionally, the study looks at the determinants of performing this habit.

## Methods

The current study was a cross sectional observational study conducted in Outpatient Gynecology Clinic of Women Health Hospital, Faculty of Medicine, Assiut University, Assiut, Egypt during the period from May 2010 through August 2011. All married women who presented to the clinic and proved to have one of the three common types of vulvovaginits (BV, candidal vulvovaginitis, trichomonus vulvovaginitis ) were invited to participate in the study. We did exclude women who were had any type of vaginal bleeding, those with undiagnosed abnormal vaginal discharge, had undetermined type of infection, those who refused to participate in the study. The non-interventional nature of the study as well as its confidentiality was explained to the participants. Oral consent has been obtained from all participants. The study protocol had been approved by the Assiut Medical School Ethical Review Board.

The diagnosis of vulvovaginits was defined as follows: BV was diagnosed when at least three of the following four criteria (Amsel’s criteria) were positive: presence of a thin homogeneous discharge, vaginal pH ≥ 4.5, positive whiff test, and the presence of clue cells in the wet mount. Vaginal pH was measured during the gynecological examination, using a colorimetric tape put in contact with the vaginal wall for 1 minute. For the amine-odor (whiff test) to be performed, two drops of 10% potassium hydroxide were added to a fraction of the discharge obtained from the posterior vaginal fornix. The test was considered positive when the characteristic fishy odor was detected. A wet-mount specimen was analyzed for the presence of clue cells under 40 × magnification [16]. Vulvovaginal candidiasis was diagnosed by patient's history, clinical features (typical odorless thick white discharge), vaginal pH(< 4.5), and direct microscopy (wet mount with saline solution and potassium hydroxide) [17,18]. Trichomonal vaginalis vaginitis was suggested by clinical history and confirmed by finding the characteristic motile flagellates in a wet mount preparation using physiologic saline and a drop of vaginal fluid on a slide covered with a cover slip [19].

Vaginal douching is defined as having any type of intentional introduction of a woman’s liquid solution inside the vagina using the hand, water jet or a pumping instrument [1,2].

The study included an interview administered questionnaire that was written in Arabic by the investigators. Pilot testing of the questionnaire was done and the final version was validated on eligible participants to determine whether it was acceptable, simple and readily understood. The questionnaire has been administered by trained clinic nurse and in-private room to ensure women freedom to express their real attitudes. The questionnaire consists of 29 short questions asking about demographic characteristics, obstetric history, and history of vaginal infections; whether the participant was regularly performing of VD and the purpose, frequency and the technique used for VD. The questionnaire were also asked about the history of occurrence of certain reproductive health hazards including; PTL, ectopic pregnancy, miscarriage and PID.

The sample size calculation was based upon the primary outcome, history of preterm labor. Previous studies reported that the general incidence of preterm labor was around 12-13% [20]. Using two sided chi-square (χ2) test with α of 0.05, a minimum sample size of at least 441 in the two groups (taking a ratio of 1:3 unexposed to exposed), this will give 110 patients in non-douching group versus 331 in the douching group, using 80% power to detect doubling the incidence of preterm labor between the 2 groups. {i.e. 13.0% in the non-douching group vs. 26.0% in the douching group} [odds ratio of 2.35] (Epi-info™, CDC, USA, 2008).

The data collected were entered on Microsoft access data base to be analyzed using Statistical Package for Social Science software (SPSS Inc., Chicago, version 13). Mean ± SD was used to summarize continuous data. Chi-square test was used to assess the significance of the difference between categorical variables. A p-value < 0.05 was considered statistically significant.

## Results

**Figure 1:**
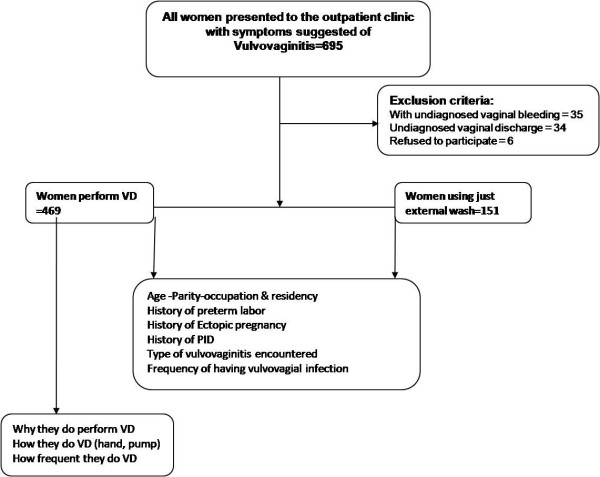
Study flowchart.

Six hundred and ninety five women with vulvovagial infections were approached for participation in the study. Of them, 75 were excluded because of not conforming to the eligibility criteria. This left 620 women who fell into two groups according whether or not they were performing VD. The current study showed that 469 of our study population (73%) were performing VD (Figure 1). Table 1, describes the scocio-demographic characteristics of women included in the study. The mean age of the participants was about 29 years. There were no significant differences between women performing VD and those not practicing as regard the occupation or the number of living children. The majority of women (88.9%) who were having the habit of performing VD were living in rural and semi urban areas.

**Table 1:** Socio-demographic data of vaginal douching practice

	***Douching (n=469)***	***Non-douching (n=151)***	***p- value***
**Age (mean±S.D)**	29.41±9.372	29.68±8.740	0.754
**N .of living children (mean± S.D)**	2.15±1.98	2.32±1.68	0.328
**N .of males (mean± S.D)**	1.06±1.23	1.09±1.02	0.766
**N of females (mean± S.D)**	1.14±1.18	1.25±1.07	0.297
**Occupation**			
**-** house wife n(%)	140 (29.9)	51 (33.8)	0.364
- Working n(%)	329 (70.1)	100 (66.2)	
**Residence**			
- Urban n(%)	52 (11.1)	20 (13.2)	0.017
- Semi-urban n(%)	253 (53.9)	97 (64.2)	
- Rural n(%)	164 (35.0)	34 (22.5)	

Douching: mean women who are performing internal vaginal douching by fingers or pumps.

Non-douching: women who were not performing any internal vaginal douching.

The causes behind performing VD were summarized in Table 2. Considering internal cleaning as a religious obligation after menstruation and sexual intercourse and as a method of intense cleaning were the most commonly given answers in 88.9% and 80.6% of women; respectively.

**Table 2:** Causes of vaginal douching from the participant point of view

**Causes of vaginal douching**	**Number (%)**
**Religious duty**	417(88.9)
**Personal cleaning**	378(80.6)
**Method of contraception**	33(7.0)
**Traditional habit**	43(9.2)
**Others**	15(3.2)

Percentages count more than 100% because some participants point to more than one cause to have vaginal douching.

Table 3: gives the reproductive health hazards associated with VD. Women who perform this habit reported having a higher incidence of PTL than those who do not (OR=1.75- CI= 1.1-3.0) Vaginal douching practice was also significantly associated with higher percentage of women having a history of PID (OR = 2.4- CI= 1.2-4.9). No statistically significant differences were found as regard history of ectopic pregnancy or number of previous abortions. Furthermore, when we stratify participants to those who were using the hand or a pump to push the liquid solution inside the vagina, the incidence became significantly higher among pump users as regards ectopic pregnancy and PID (p=0.005 and 0.005; respectively). On the other hand, there were no significant difference between hand and pump users as regard history of PTL.

**Table 3:** Reproductive health hazard in women performing vaginal douching

	**Douching (n=469)**	**Non-douching (n=151)**	**Odds ratio (CI)**	**P value**
**Preterm labor (n%)**	90 (19.2)	18 (11.9)	1.75 (1.1-3.0)	0.048
**Ectopic pregnancy (n%)**	18 (3.8)	4 (2.6)	1.47 (0.49-4.4)	0.345
**Pelvic inflammatory disease (n%)**	62 (13.2)	9 (6.0)	2.4 (1.2-4.9)	0.008
**N .of abortions (mean ± S.D)**	0.71±1.95	0.61±1.43	_____	0.353

**Figure 2:**
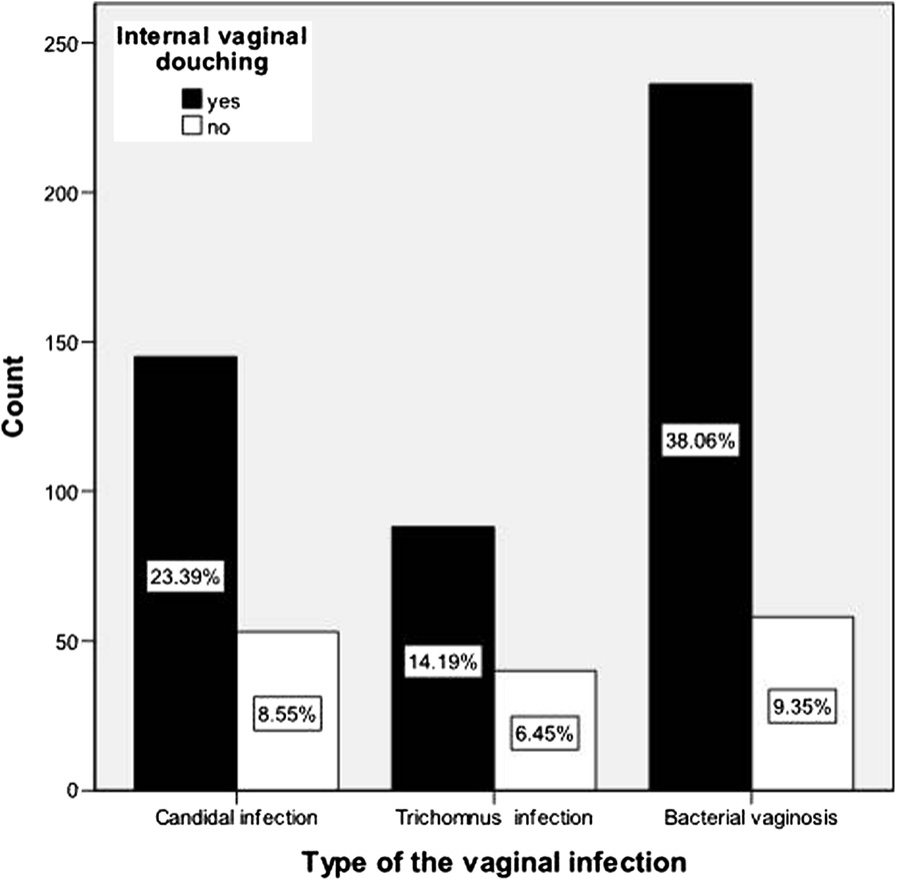
The relation between the performances of internal vaginal douching to the type of vulvovaginitis.

Bacterial vaginosis was the most common type of vaginal infection encountered followed by vaginal candidiasis and trichomanis vaginalis vaginitis (47.4, 31.9 and 20.6,%, respectively). However, there is no statistical correlation between the type of infection encountered and the performance of VD (Figure 2). When we tried to correlate the relation between VD and the frequency of having vaginal infection; the infections were encountered more than once per month in about 11% of women who had VD compared to 4% in those who had not (p=0.008).

## Discussion

Placing of a liquid solution in the vagina is a common practice in many societies all over the world. It is common among black American and it is common practice in many of the developing countries especially those with Islamic predominant population [15]. The current study is evaluating the implications of VD practice among married women in Assiut, Egypt. Our study demonstrated that about three quarters (73%) of married females presented with different types of vulvovaginal infections were of the habit of performing VD. This is the same prevalence of VD habit (about 70%) was previously reported among all attendees of the outpatient clinic of the same Hospital [8]. This figure is much more than what has been reported in the USA, about 30-48% of black Americans and 12-27% of white Americans were performing VD [4]. Different culture, traditions and religious backgrounds may be behind the above discrepancy.

Why does women douche her vagina is a matter of variance between different societies? Our study demonstrated that thinking the practice as a religious obligation is the most prominent reason, followed by considering VD as a part of personal cleaning. Studies in the USA had point to personal hygiene and feeling clean and fresh as predominant reasons.

Reproductive health hazards are the main concern behind performing VD. Bacterial vaginosis was the commonest type of vaginal infection encountered in the present cohort. Our results conform with previous reports that pointed to increased risk of BV in women performering VD compared with those not performing this habit [11,21].

The present study showed that users of VD were at a higher risk of PTL compared to those who did not practice this habit (p=0.048). These results was in accordance to the cohort study done in the USA [11,12]. The same conclusion has been obtained from a previous randomized controlled trial (RCT) done in New York as once per week VD was associated with a four-fold increase in PTL [22].

Pelvic inflammatory disease was another important reproductive health hazard that was more common among women who were practicing VD. We point to high prevalence of PID in our society; in 45% of those practiced VD as compared to 22% of those did not. This results is in accordance to the meta-analysis of published studies from 1965 to 1995 which did prove that VD increased the risk for PID by 73% [7]. Something that is may be linked to increase incidence of tubal factor infertility on our society. However, this suggested correlation needs a bigger study sample to be proven. We have also demonstrated that using internal pump for pushing cleansing solution inside the vagina carries significant higher risk than those just using their hands to get the liquid inside. The association between VD and PID had been confirmed by an RCT by Ness and colleagues 2005 [23]. These results let the Center of Disease Control (CDC) to put an obligation for commercial douche boxes to contain warnings about the association between vaginal douching and PID.

The current questionnaire reported high incidence of ectopic pregnancy among douching users compared to none users of this habit. However, the difference did not reach a significant value. This is contradicting what has been founded in the literature that VD increased risk of ectopic pregnancy by 76% [2]. The difference may be attributed to the difference between the prevalence of STIs in different societies.

The practice of VD has deep roots in the community of Upper Egypt. Asking women about the reasons why they had used this practice, discovered many misbeliefs that washing the internal parts of their reproductive outlet is a necessary part of the religious obligatory purification after sexual intercourse or menses (Ghusl) (in about 88% of the current study participants). Revising the religious roles in Islam showed that Ghusl does not necessitate by any mean any type of internal VD. Moreover, there are other misunderstandings about VD in higher class population, considering this kind of internal cleaning as a type of intense hygiene that keeping them unsoiled and fresh (80.6%). Other less educated women think it could be a method of contraception. They were unaware about the possible reproductive health hazards of this practice.

Asking about the origin of learning this practice,showed that VD were performed according to an advise from women's mothers, friends, neighbors or occasionally from a nurse or a doctor. The above influences point to the importance of campaigns of information and education tackling these areas in any effort planned to limit this hazardous practice.

## Conclusion

In conclusion, VD is a common practice among married Egyptian women and has its traditional and religious backgrounds. Vaginal douching does increase the incidence of PTL and PID. Awareness campaigns are needed among women and health providers about health hazards of this incorrect practice to limit its use.

## Competing interests

The authors declare that they have no competing interests.

## Authors’ contributions

OS was the founder of the research idea,designed the data collection sheet,carried out the statistical analysis, drafted the manuscript. AY shared in formulation of the research question, designed the data collection sheet, drafted the manuscript. MK Carried out data collection and data entry. SM shared in formulation of the research question,designed the data collection sheet, drafted the manuscript, All authors read and approved the final manuscript.

## Pre-publication history

The pre-publication history for this paper can be accessed here:

http://www.biomedcentral.com/1472-6874/13/23/prepub
